# Penile Fracture: Delayed Presentation, Primary Urethral Repair and Satisfactory Outcome

**DOI:** 10.1155/2019/1456914

**Published:** 2019-10-07

**Authors:** Bashir Yunusa, Kalamon Wullie, Soeghen E. Willie, Solomane Konneh, Swaliho Sherriff, Ayun Cassell, Edit Ikpi

**Affiliations:** ^1^Department of Surgery, Liberia College of Physicians and Surgeons, Monrovia, Liberia; ^2^Département d'Urologie et d'Andrologie, Hôpital Général de Grand Yoff, Dakar, Senegal

## Abstract

Penile fracture is a relatively rare condition warranting emergency intervention. The commonest etiological factor remains coital activities, which explains why it is being underreported. Presentation is usually delayed and up to 38% of cases present with associated urethral injury. Prompt surgical intervention and primary urethral repair are associated with a good outcome. We present a 30-year-old male with unilateral penile fracture and associated urethral injury following sexual intercourse.

## 1. Introduction

Penile fracture is defined as the rupture of the tunica albuginea of the corpus cavernosum caused by blunt trauma to the erect penis [1]. The condition is relatively rare and under reported [2], but its occurrence requires emergency intervention. There are several etiological factors, but blunt trauma from coitus accounts for most causes [3]. Others include falls, forceful manipulation or masturbation [4, 5] or rolling over an erect penis [6], and Penile curvature (chordae) [7]. Penile fractures usually present with a ‘popping' sound with concomitant sudden swelling and ecchymosis of the penis followed by rapid detumescence [2]. A significant number of cases come with associated urethral injury, ranging from 3% to 38% in different literatures [8, 9]. Before now, we do not know whether delay in presentation or surgical intervention may affect the outcome of repair or risk recurrence [10].

We present here a case of 30-years-old man who presented two weeks after he sustained penile fracture from rigorous sexual intercourse to the ER of John F. Kennedy Medical Centre, Monrovia, Liberia.

A 30-years-old Liberian male presented with painful swollen penis following sexual intercourse for 2 weeks. Patient was involved in sexual activity in the male dominant position when his penis slipped out and hit the inner thigh of his partner with immediate excruciating pain along the shaft of his penis, slow detumescence ranging 3–5 minutes, swelling of the right side of his penis, and left curvature. He denied the use of sex enhancing drugs and did not admit penile curvature prior to the trauma. He presented within 2 weeks to the emergency room following increasing painful micturition and hematuria where he had successful catheterization prior to urological review.

All other examinations were normal except the area of pathology. The distal half of the penis, including the glans, was tilted to the left, the penile shaft appeared edematous without apparent discoloration. There was neither associated scrotal nor perineal swelling, urethral catheter in situ draining bloody urine. There was tenderness on palpation of the penile shaft with the point of maximum tenderness on the right mid-third of the shaft. He was diagnosed of penile fracture with possible urethral injury and prepared for surgery.

### 1.1. Intraoperative Findings

A degloving incision was made through the skin, the Dartos fascia found intact and then separated, and the deep penile (Buck) fascia was found to have a bulging hematoma on the right mid shaft and an approximate 4 cm transverse defect on the ventral surface of the right tunica albuginea with complete urethral defect at the same spot were seen. Tunical defect was repair in two layers with PDS 3/0 suture and the urethra spatulated and anastomosed with vicryl 3/0 over the indwelling catheter. The Buck's and Dartos fascia and the penile skin closed in layers in a simple interrupted fashion. Saline induced erection demonstrated leak free repair. Patient admitted having morning tumescence. Catheter was removed after 2 weeks of repair and rigid cystoscopy revealed intact urethra with visible suture lines.

## 2. Discussion

A penile fracture typically occurs in the setting of blunt penile trauma to the erect penis, most often during sexual intercourse or masturbation. The characteristic symptoms of penile fracture are a “snapping” or “popping” sound, penile pain, and immediate detumescence followed by ecchymosis and swelling of the penile shaft [11]. Distinguishing penile ecchymosis from a true penile fracture, or a rupture through the tunical albuginea, can be a challenging clinical conundrum, most especially in patients like ours, who presented up to 2 weeks after the trauma and denied the typical popping sounds, with a relatively long time of 3–5 minutes for penile collapse but admitted painful urine and hematuria.

Physical examination findings may vary significantly in patients with a history suggestive of penile fracture, especially in late presentation, and the severity of the penile ecchymosis frequently does not correlate with the presence or absence of tunical rupture. History and physical examination may be inaccurate in 15% of patients with a suspected penile fracture [11], as non-sex related rupture has also been described [12]; but the typical history of slipping and striking the perineum made us suspect the fracture. With the history of late presentation, we did not find ultrasound or retrograde urethrography (RUG), necessary, since, further delay can increase risk of infection and probable suture dehiscence [13, 14].

**Figure 1:**
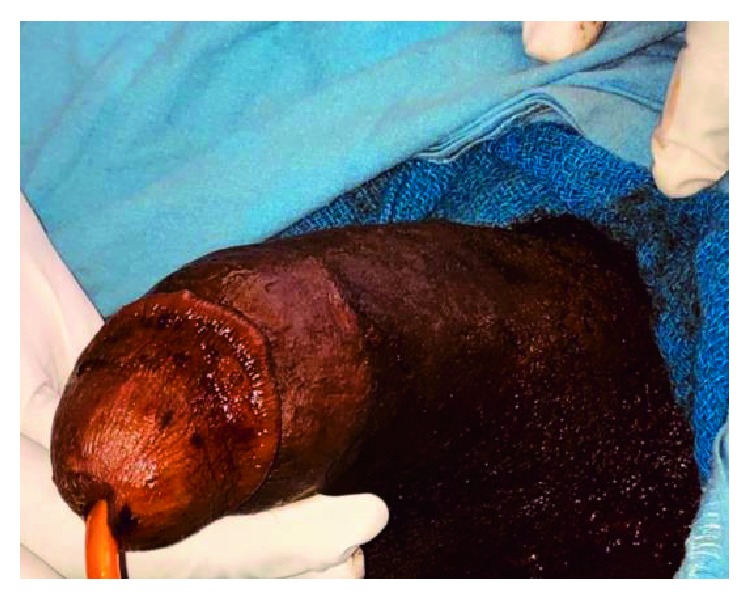
The penis preoperatively, with left curvature and right mid shaft swelling.

The patient presented with swollen right sides and deviation of the phallus to the left as shown in (Figure 1), suggestive of ipsilateral/unilateral fracture. Unilateral fractures are common in 89.7% of cases in some series and very rarely bilateral cases in up to 1.9% [14]. Where isolated fractures occur, right fracture is more frequently documented, up to 53.2% than left 45.2% [15]. With unilateral rupture suspected due to the curvature to the left, painful hematuria and no history of curvature before trauma, a carvenosogram could have been done to rule out bilateral rupture. However, due to delayed presentation, suspected increased risk of infection and absent radiological facilities and expertise in our centre, we did not consider the study. RUG would have shown partial or complete disruption of the urethra. Although, complete urethral disruption, would have been more associated with bilateral rupture, prior catheterization might have caused the complete injury seen intraoperatively.

Penile fractures are common among young adults, with average age of 38 years, who are sexually very active, with aggressive sexual behaviors, and the tendency to use sex enhancing drugs for various reasons as described by Tang et al. [15]; further describing summer seasons with highest admission due to penile fracture, when young adults are more likely to have high sexual contacts. But, in Africa, where these adults may be close to or under the care of the parents, may feel ashamed to disclose the ailment to other family members or even hospital staff, leading to delayed presentation; and the concomitant lack of urologist may lead to delayed repair due to time taken for referrals.

**Figure 2:**
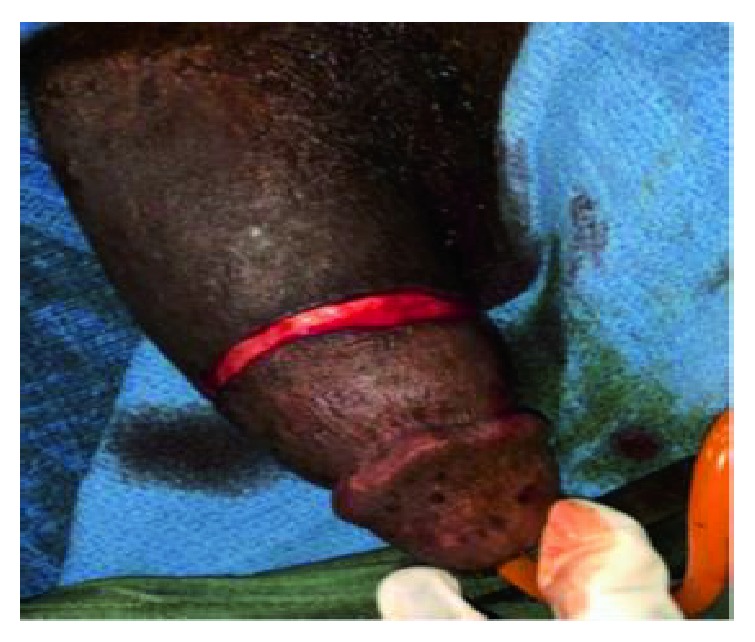
Degloving incision at the beginning of surgery.

**Figure 3:**
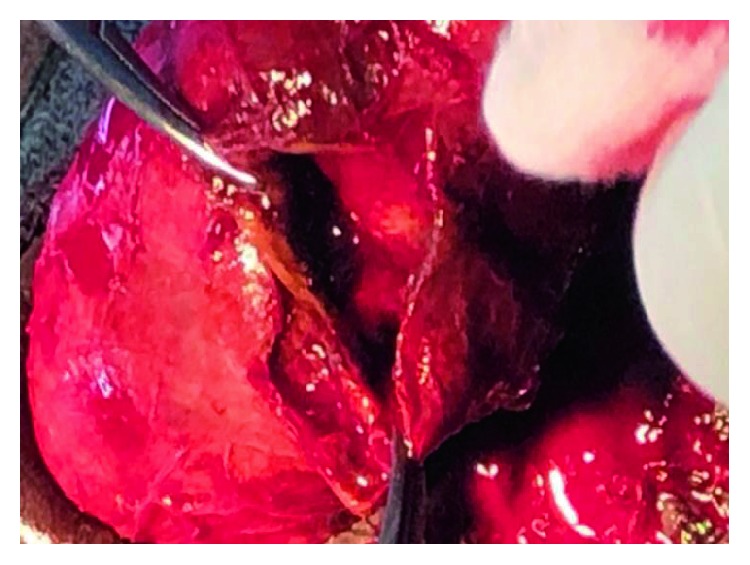
The right exposed tunica albuginea with a 4 cm transverse defect.

**Figure 4:**
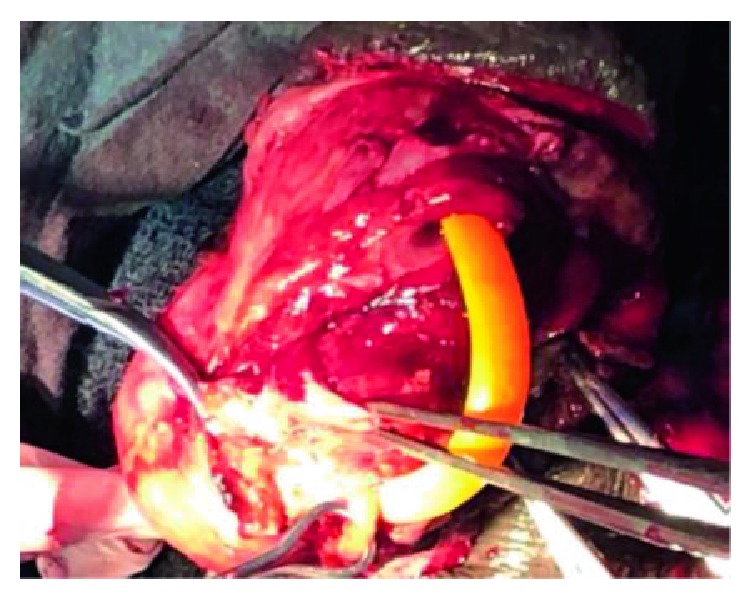
The completely disrupted urethra and part of the exposed erectile bodies.

**Figure 5:**
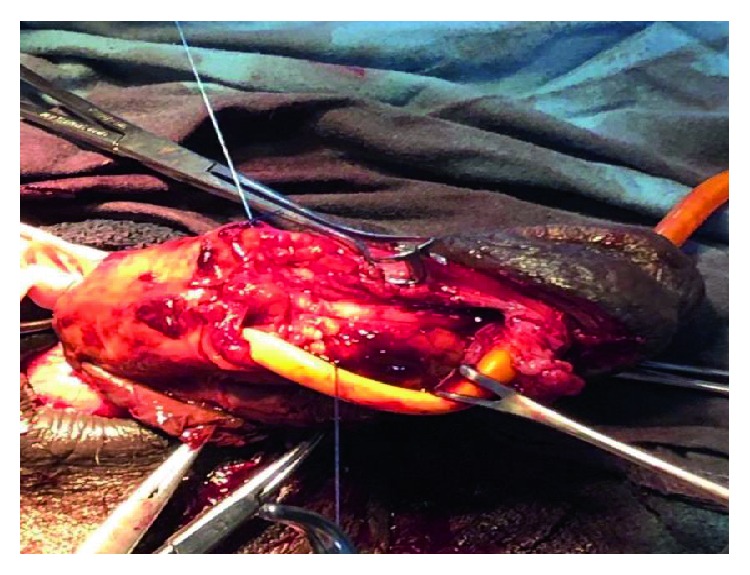
Repaired right tunica albuginea first layer.

**Figure 6:**
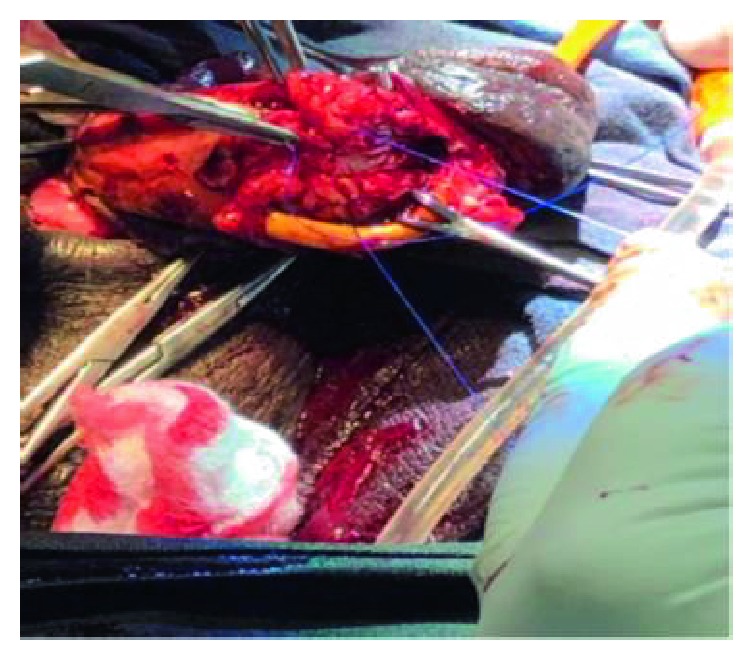
Repair of tunica albuginea of the right corpus Cavernosum, second layer.

Subcoronal skin incision [14] gives good access for degloving and is cosmetically appealing as shown in (Figure 2). Our findings included bulging hematoma on the right Bucks' fascia and a 4 cm transverse tear on the tunica mid shaft (Figure 3) with complete disruption of the urethra at the same level (Figure 4). Associated urethral injuries are documented in up to 38% of cases [15, 16]. The defect was repaired in two layers with PDS 3/0 suture (Figures 5 and 6). The approach was based on knowledge of the clinical application of the two layered anatomy of the tunica albuginea of the corpus caversosum as reported by Hsu et al. [17]. Thus, the repair of the outer longitudinal layer was considered a determinant factor of the surgical success in this tunical surgery regardless of the strength of the suture, even with our choice of PDS suture [17].

**Figure 7:**
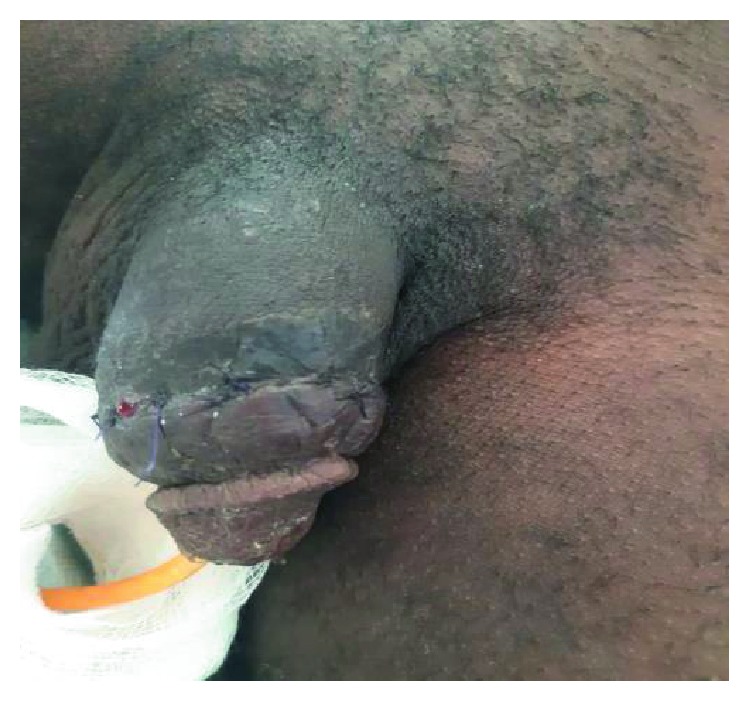
Penile straightened after repair.

**Figure 8:**
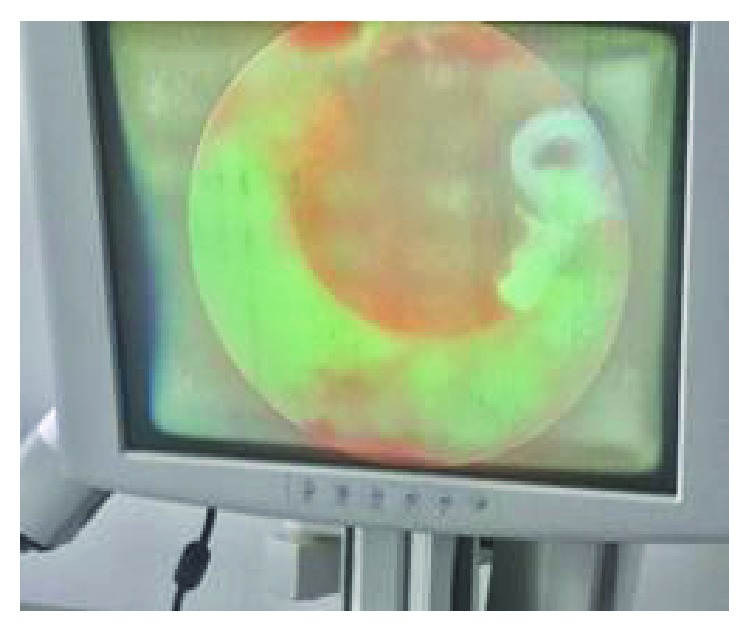
Rigid cystoscopy revealed intact urethra with some yet to be absorbed vicryl.

The urethra was repaired primarily, spatulated, and anastomosed with vicryl 3/0 over the indwelling catheter with continuous suture [18, 14]; although interrupted suture repairs are most documented [19]. The postoperative outcome was satisfactory within two weeks; as primary repair has been proven to have a good outcome in some reports [16]. Hsu and colleagues documented curvature correction after some series of repair (7), but we could not find reasons for subsequent repair of curvature, as there was no previous history of curvature or demonstrable postoperative curvature. We obtained a good outcome with continuous sutures on top of delayed presentation. Delayed presentation and or repair have been documented to have satisfactory outcome. There was immediate straightening of the penis after the repairs (Figure 7) and an intact urethra was demonstrated by rigid cystoscopy (Figure 8) two weeks postoperatively.

## 3. Conclusion

Penile fracture is either rare or under reported, patients in our resource depleted environment tend to conceal or delay presentation, but delayed presentations and primary urethral repair do have satisfactory outcome.
